# Fluoroquinolone Prophylaxis Reduces Intensive Care Unit Admission and *Clostridioides difficile* Rates in Acute Myeloid Leukemia Induction Chemotherapy

**DOI:** 10.1002/jha2.70225

**Published:** 2026-02-15

**Authors:** Charles J. Weeks, Sujith Abbagoni, Faizan Boghani, Priyanka Menon, Matthew Gold, Chandini Kannan, Ozal Ismayilov, Mark Dalgetty, Anvay Shah, Lydia Delay, Heeju Lim, Mohammad Mian, Andrea Clarke, Locke Bryan, Jorge Cortes, Anand Jillella, Vamsi Kota

**Affiliations:** ^1^ Medical College of Georgia Augusta Georgia USA; ^2^ Department of Internal Medicine Wellstar‐MCG Hospital Augusta Georgia USA; ^3^ Georgia Cancer Center Augusta Georgia USA

**Keywords:** acute myeloid leukemia, bloodstream Infections, *Clostridioides difficile*, fluoroquinolones, induction chemotherapy, intensive care unit

## Abstract

**Background:**

Patients with acute myeloid leukemia (AML) undergoing induction chemotherapy are at high‐risk of bloodstream infections, including central line‐associated bloodstream infections (CLABSI) or mucosal barrier injury‐related infections (MBI‐LCBI). The impact of fluoroquinolone (FQ) prophylaxis on MBI‐LCBI, *Clostridioides difficile*, and intensive care unit (ICU) outcomes in this population remains unclear.

**Methods:**

We retrospectively reviewed adults with AML receiving induction chemotherapy between 2010 and 2022. Patients were grouped by receipt of FQ prophylaxis. Primary outcomes were incidence of CLABSI, and MBI‐LCBI. Secondary outcomes included neutropenic fever, ICU admission, length of stay, and *C. difficile* infection. Microbiologic patterns were analyzed by time and prophylaxis status.

**Results:**

Among 195 patients, 88 (45.1%) received FQ prophylaxis. CLABSI rates were 8.0% with prophylaxis versus 15.0% without (*p* = 0.132), and MBI‐LCBI rates were 11.4% versus 15.0% (*p* = 0.463). ICU admission was significantly lower with prophylaxis (10.2% vs. 31.8%**;** *p* < 0.001). *C. difficile* infection was also less frequent among those receiving prophylaxis (5.7% vs. 17.8%; *p* = 0.012). Neutropenic fever rates were similar between groups. CLABSI isolates were primarily skin flora and staphylococcal species, whereas MBI‐LCBI isolates were exclusively enteric flora, increasingly involving Gram‐negative rods over time. *Pseudomonas* infections were more frequent among patients without prophylaxis.

**Conclusions:**

In this cohort, FQ prophylaxis did not significantly reduce bloodstream infections but was associated with fewer ICU admissions and lower rates of *C. difficile* infection. These findings further support the use of FQ prophylaxis with minimal concern for exacerbation of *C. difficile* rates. It further highlights ongoing risk from enteric translocation despite prophylaxis.

## 1 Introduction

Acute myeloid leukemia (AML), the most common hematologic malignancy, is driven by clonal expansion of undifferentiated myeloblasts, typically arising from de novo mutations in hematopoietic genes [1, 2]. Standard treatment includes 7 + 3 induction and high‐dose cytarabine (HiDAC) consolidation chemotherapy, both requiring central venous access [3, 4]. These regimens induce profound neutropenia and immunosuppression, increasing susceptibility to serious infections, including central line‐associated bloodstream infections (CLABSI) [4].

CLABSI is defined as a laboratory‐confirmed bloodstream infection in a patient with a central line in place > 48 h, without another infection source [5]. It can lead to sepsis, organ dysfunction, and ICU admission [6, 7, 8]. Febrile neutropenia, another common complication, similarly carries a high‐risk of severe infection. Prophylactic fluoroquinolones (FQ) prevent the onset of febrile neutropenia and CLABSI in patients with AML [9] though concerns remain with their association with increased *Clostridioides difficile* infection [10, 11]. Importantly, many bloodstream infections attributed to CLABSI may actually result from mucosal barrier injury (MBI) during prolonged neutropenia, which facilitates translocation of enteric organisms into the bloodstream [12, 13, 14]. The highly sensitive definition of CLABSI may overattribute bloodstream infections to the catheter [15, 16]. To address this, MBI‐LCBI recognizes LCBIs that originate from gastrointestinal sources with intestinal organisms [16, 17, 18].

Although guidelines to prevent the onset of CLABSI and MBI‐associated bloodstream infections have been established, the effects of prophylactic measures on CLABSI development and incidence of MBI‐LCBI events in patients with AML remain unclear [1, 14]. FQ prophylaxis has been found to improve mortality [19] and be highly effective for the prevention of CLABSI and neutropenic fever with no difference in *C. difficile* rates in autologous transplants [20] and AML induction [21]. The objective for this study is to characterize the effects of antibiotic prophylaxis on the incidence of MBI‐LCBI, *C. difficile* infection, CLABSI, neutropenic fever, and ICU admission for patients with AML. To the best of our knowledge, no studies have evaluated whether FQ prophylaxis impacts MBI‐LCBI or ICU admission in patients undergoing induction therapy for AML.

## 2 Methods

### 2.1 Study Design and Setting

We conducted a retrospective cohort study evaluating adult patients with AML who underwent induction chemotherapy between January 1, 2010, and December 31, 2022, in Augusta, Georgia, and associated with the Georgia Cancer Center. The study was approved by the institutional review board at the Medical College of Georgia with a waiver of informed consent due to its retrospective nature.

### 2.2 Patient Population

Patients aged 18 years or older diagnosed with AML and admitted for induction chemotherapy were eligible for inclusion. Exclusion criteria included insufficient clinical data or absence of central venous catheter placement during hospitalization. Patients were categorized based on whether they received FQ prophylaxis during their induction chemotherapy admission. The decision to administer FQ prophylaxis was at the discretion of treating physicians, in line with evolving institutional protocols over the study period.

### 2.3 Definitions

LCBSI was defined as isolation of a pathogen from one or more blood cultures in a patient with clinical signs of infection [5]. CLABSI was defined as a laboratory‐confirmed bloodstream infection in a patient who had a central venous catheter in place for more than 48 h, with no other identifiable source of infection [5]. MBI‐LCBI was defined as an LCBSI associated with gastrointestinal sources with intestinal organisms such as viridans group streptococci and yeast in the setting of severe neutropenia (absolute neutrophil count [ANC] < 0.5 × 10^9^/L for ≥ 2 days within a 7‐day window around the date of the positive culture) and absence of another infectious source [16, 17, 18]. As all of our patients had central lines placed for induction chemotherapy, the LCBI were either a CLABSI or MBI‐LCBI.

FQ Prophylaxis was defined as receipt of either levofloxacin or ciprofloxacin during hospitalization for AML induction chemotherapy prior to the onset of neutropenic fever or positive blood cultures. Viral prophylaxis referred to treatment with acyclovir over the same period and fungal prophylaxis referred to treatment with an azole or micafungin. Neutropenic Fever was defined as a single oral temperature ≥ 38.3°C in the presence of an ANC < 0.5 × 10^9^/L [22]. ICU Admission was defined as transfer to an ICU for management of medical complications during the same hospitalization. *C. difficile* Infection was defined as the presence of diarrhea and detection of *C. difficile* toxin with enzyme immunoassay following a positive nucleic acid amplification or glutamate dehydrogenase antigen test [23, 24, 25]. Total parenteral nutrition (TPN) feeding refers to whether or not the patient received parenteral nutrition at any point during induction. In this study, 60‐day mortality refers to death of the patient by any cause within 60 days of the start of induction chemotherapy.

Gram‐negative rods included Klebsiella pneumoniae, Escherichia coli, Pseudomonas aeruginosa, Enterobacter cloacae, and Campylobacter urolyticus. Gram‐positive organisms encompassed Staphylococcus aureus (methicillin‐susceptible and methicillin‐resistant) (MSSA and MRSA), coagulase‐negative staphylococci, Staphylococcus warneri, Rothia mucilaginosa, Streptococcus mitis, Streptococcus viridans, and Enterococcus faecium. Fungal pathogens included Candida species. Site of origin was defined as enteric flora (e.g., K. pneumoniae, E. coli, E. faecium, Streptococcus spp., Candida), skin flora (e.g., staphylococci), or Pseudomonas aeruginosa as its own distinct group due to its environmental reservoir and clinical relevance. Common pathogen categories included Staphylococcal species, Gram‐negative rods, opportunistic pathogens (including Rothia, Streptococcus spp., Campylobacter, and Candida), and Enterococci.

### 2.4 Data Collection

Data were abstracted from electronic medical records, including demographics, AML diagnosis dates, fungal prophylaxis, viral prophylaxis, TPN use, microbiology results, ICU admissions, and outcomes. Microbiologic data were extracted to determine causative pathogens, classified by Gram stain, site of origin, and common pathogen category. Mortality data were collected from the EMR if patients died while admitted at Wellstar‐MCG Hospital, but were otherwise collected from obituary sources. The analysis used the date of AML diagnosis as the reference point for categorizing the study period into two intervals: 2010–2016 and 2017–2022.

### 2.5 Outcomes

The primary outcomes were the incidence of CLABSI and MBI‐LCBI during induction chemotherapy. Secondary outcomes included neutropenic fever, ICU admission, ICU length of stay (LOS), total hospital LOS, 60‐day mortality, and incidence of *C. difficile* infection.

### 2.6 Statistical Analysis

Baseline characteristics were compared between groups using chi‐square tests for categorical variables and analysis of variance (ANOVA) for continuous variables. Continuous data was reported as median and interquartile range (IQR). Associations between FQ prophylaxis and outcomes were evaluated using chi‐square tests or Fisher's exact tests as appropriate. Odds ratios (OR) were calculated for categorical outcomes. ANOVA was used to compare continuous variables when appropriate. A multiple logistic regression (MLR) was done to evaluate the effects of possible contributors on the primary outcomes and ICU admission. Logistic regression was selected to allow multivariable adjustment for clinically relevant covariates, and OR were reported for consistency across unadjusted and adjusted analyses. A *p*‐value < 0.05 was considered statistically significant. While *p*‐values are reported for unadjusted analyses, inference for multivariable models was based on confidence intervals. Area under the receiver operating characteristic curve (AUC) was used to assess model discrimination, defined as the probability that the model assigns a higher predicted risk to a patient who experienced the outcome than to a patient who did not. An AUC of 0.5 indicates no discriminatory ability, whereas an AUC of 1.0 indicates perfect discrimination. Statistical analyses were conducted using SPSS Version 29 and GraphPad Prism Version 10.

## 3 Results

### 3.1 Baseline Characteristics

A total of 195 patients undergoing induction chemotherapy for AML were included in the analysis, and 88 (45.1%) received FQ prophylaxis, and 107 (54.9%) did not. The median duration of FQ prophylaxis was 17 (IQR 10–22) days. A total of 23 patients were diagnosed with a CLABSI and 26 with MBI‐LCBI. The median age of the entire cohort was 58.8 years (IQR 43.9–68.2) with no significant difference between those who received FQ prophylaxis or not (*p* = 0.704). Less than half of the patients overall were over 60 years of age (48.2%) with no significant difference between those who received FQ prophylaxis or not (*p* = 0.756). Females comprised 48% of the cohort, with no significant difference between those who received FQ prophylaxis or not (*p* = 0.756). (Table 1).

**TABLE 1:** Sample baseline characteristics and microbiological data.

Outcome	FQ prophylaxis (*n* = 88)	No FQ prophylaxis (*n* = 107)	Odds ratio (OR)	*p*‐value
Number of females (% female)	44 (50%)	50 (46.7%)	−	0.756^a^
Median age and IQR (years)	58.2 (45.5–68.5)	59.0 (42.4–67.4)	−	0.704^b^
Proportion age > 60 years	44 (50.0%)	50 (46.7%)	−	0.756^a^
Median duration of FQ prophylaxis and IQR (days)	17 (10–22)	−	−	−
Neutropenic fever	66 (75.0%)	90 (84.1%)	0.57	0.113^a^
ICU admission	9 (10.2%)	34 (31.8%)	0.25	< 0.001^a^
*Clostridium difficile* infection	5 (5.7%)	19 (17.8%)	0.28	0.012^a^
MBI‐LCBI	10 (11.4%)	16 (15.0%)	0.73	0.463^a^
True CLABSI	7 (8.0%)	16 (15.0%)	0.49	0.132^a^
LCBSI (overall)	17 (19.3%)	32 (29.9%)	0.56	0.090^a^
60‐Day mortality	3 (3.4%)	5 (5.7%)	0.58	0.660^a^
Viral prophylaxis	88 (100%)	102 (95.3%)	**∞** ^c^	0.058^a^
Fungal prophylaxis	85 (96.6%)	99 (92.5%)	2.36	0.361^a^
TPN feeding	4 (4.5%)	10 (9.3%)	0.46	0.268^a^
Median hospital LOS and IQR (days)	30 (25–37)	30 (27–37)	−	0.220^b^
Median ICU LOS and IQR(days) (among ICU patients)	4 (2‐5)	5 (3–9)	−	0.085^b^
Median time from induction to ICU admission and IQR (days) (among ICU patients)	19 (15–23)	11 (4.5–19.75)	−	0.187^b^
Median time from induction to febrile neutropenia and IQR (days)	12.5 (9–20.5)	7 (3–10)	−	< 0.001^b^
FQ‐resistant microbes	10 (11.4%)	7 (6.5%)	1.84	0.015^a^
ESBL microbes	0 (0%)	0 (0%)	−	^−^

^a^ *p*‐values from chi‐square test.

^b^ *p*‐values from ANOVA.

^c^ OR undefined due to zero cells (perfect separation).

### 3.2 Outcomes by Prophylaxis Group

All patients in the FQ group received viral prophylaxis (100%), compared to 95.3% in the non‐FQ group (p = 0.058). Similarly, fungal prophylaxis was administered to 96.6% of patients who received FQ and 92.5% of those who did not (OR = 2.36, p = 0.361). TPN was administered to 4.5% of patients who received FQ prophylaxis and 9.3% of those who did not (OR = 0.46, p = 0.268). Among patients who received FQ prophylaxis, 17 of 88 (19.3%) developed a LCBI compared to 32 of 107 (29.9%) among those without prophylaxis, though this difference was not statistically significant (*χ* ^2^ = 2.877, *p* = 0.090; OR = 0.56). CLABSI occurred in 8.0% (7/88) of the prophylaxis group versus 15.0% (16/107) without prophylaxis, also without statistical significance (*χ* ^2^ = 2.273, *p* = 0.132; OR = 0.49). MBI‐LCBI was identified in 11.4% (10/88) of patients with prophylaxis and in 15.0% (16/107) of patients without prophylaxis (*χ* ^2^ = 0.538, *p* = 0.463; OR = 0.73). The median hospital LOS was 30 days (IQR 25–37) overall, without significant difference between prophylaxis groups (*p* = 0.220). Although patients receiving FQ prophylaxis had a lower 60‐day mortality rate (3.4%) compared to those without prophylaxis (5.7%), this difference was not statistically significant (OR = 0.58, *p* = 0.660) (Table 1).

The incidence of *C. difficile* infection was significantly lower among patients receiving FQ prophylaxis (5.7%, 5/88) compared with those who did not (17.8%, 19/107) (*χ* ^2^ = 6.384, *p* = 0.012; OR = 0.28). Neutropenic fever occurred in 75.0% (66/88) of patients receiving prophylaxis and 84.1% (90/107) without prophylaxis, which was not a statistically significant difference (*χ* ^2^ = 2.506, *p* = 0.113; OR = 0.57). The median duration of time from induction to febrile neutropenia was significantly longer in patients who received FQ prophylaxis (12.5 days IQR 9–20.5) than those who did not (7 days IQR 3–10, *p* < 0.001). However, FQ resistant blood cultures grew in 11.4% of patients receiving prophylaxis and 6.5% without prophylaxis (*χ* ^2^ = 5.92, *p* = 0.015; OR = 1.84) (Table 1).

ICU admission was significantly less frequent in the prophylaxis group, occurring in 10.2% (9/88) compared to 31.8% (34/107) among those without prophylaxis (*χ* ^2^ = 12.686, *p *< 0.001; OR = 0.25). Among patients admitted to the ICU, median ICU LOS was 4 (IQR 2–5) days in the prophylaxis group and 5 (IQR 3–9) days in the no‐prophylaxis group (*p* = 0.085), with no statistically significant difference. The median time from induction to ICU admission was also not significantly different between groups: 19 (IQR 15–23) days for patients receiving prophylaxis versus 11 (IQR 4.5–19.75) days for those without (*p* = 0.187) (Table 1). When stratified by FQ prophylaxis status, ICU admissions among patients who received prophylaxis were most often due to hypoxia or respiratory failure (55.6%), followed by equal proportions of sepsis (22.2%) and cardiac causes (22.2%). In contrast, patients who did not receive prophylaxis were more commonly admitted for cardiac arrhythmia or observation (44.1%), with lower proportions admitted for hypoxia (29.4%) and sepsis (26.5%).

#### 3.2.1 Microbiologic Data

Among patients who developed CLABSI, mean LOS was 34.7 days (SD ± 15.1), and for those with MBI‐LCBI it was 33.2 days (SD ± 13.2). ICU admission occurred in 43 patients (22.1%) overall, including 21.7% of those with CLABSI and 23.1% of those with MBI‐LCBI (*p* = 0.986). Febrile neutropenia was observed in 91.3% of patients with CLABSI and 84.6% with MBI‐LCBI (*p* = 0.275). TPN use was observed in 7.8% of all AML patients, with 4.3% of patients in the CLABSI group and 11.5% in the MBI‐LCBI group receiving TPN. The difference in TPN exposure between groups was not statistically significant (p = 0.612). Sixty‐day mortality differed significantly across groups, occurring in 4.1% of all AML patients, but rising to 17.4% in those with CLABSI and 15.4% in those with MBI‐LCBI (*p* = 0.008) (Table S1).

Microbiologic profiles of bloodstream infections are summarized in Tables 2 and 3. Between 2010 and 2016, 9 CLABSI infections and 14 MBI‐LCBI infections were identified, while from 2017 to 2022 there were 14 CLABSI infections and 12 MBI‐LCBI infections. Among CLABSI cases overall, Gram‐positive organisms predominated in the later time period (71.4% in 2017–2022 vs. 44.4% in 2010–2016), whereas Gram‐negative organisms accounted for more than half of infections in the earlier period (55.6%). No fungal pathogens were isolated in any CLABSI cases. Staphylococcal species were the most frequent CLABSI pathogens in both eras (44.4% in 2010–2016 and 42.9% in 2017–2022), followed by Gram‐negative rods (55.6% and 21.4%, respectively). *Pseudomonas* accounted for approximately one‐third of CLABSI infections overall (Table 2).

**TABLE 2:** CLABSI and MBI‐LCBI microorganism prevalence in AML patients over time.

Variable	2010–2016 CLABSI (*n* = 9)	2017–2022 CLABSI (*n* = 14)	2010–2016 MBI‐LCBI (*n* = 14)	2017–2022 MBI‐LCBI (*n* = 12)
Gram stain				
Gram‐negative	5 (55.6%)	4 (28.6%)	3 (21.4%)	6 (50.0%)
Gram‐positive	4 (44.4%)	10 (71.4%)	11 (78.6%)	4 (33.3%)
Fungus	0 (0.0%)	0 (0.0%)	0 (0.0%)	2 (16.7%)
Site of origin				
Enteric flora	1 (11.1%)	5 (35.7%)	14 (100.0%)	12 (100.0%)
Skin flora	4 (44.4%)	5 (35.7%)	0 (0.0%)	0 (0.0%)
*Pseudomonas*	4 (44.4%)	4 (28.6%)	0 (0.0%)	0 (0.0%)
Common pathogen category				
Staphylococcal species	4 (44.4%)	6 (42.9%)	0 (0.0%)	0 (0.0%)
Gram‐negative rod	5 (55.6%)	3 (21.4%)	3 (21.4%)	6 (50.0%)
Opportunistic pathogen	0 (0.0%)	5 (35.7%)	4 (28.6%)	2 (16.7%)
Enterococci	0 (0.0%)	0 (0.0%)	7 (50.0%)	4 (33.3%)

**TABLE 3:** CLABSI and LCBI microorganism prevalence by prophylaxis status.

Variable	CLABSI prophylaxis (*n* = 7)	CLABSI no prophylaxis (*n* = 16)	MBI‐LCBI prophylaxis (*n* = 10)	MBI‐LCBI no prophylaxis (*n* = 16)
Gram stain				
Gram‐negative	2 (28.6%)	7 (43.8%)	4 (40.0%)	5 (31.3%)
Gram‐positive	5 (71.4%)	9 (56.3%)	5 (50.0%)	10 (62.5%)
Fungus	0 (0.0%)	0 (0.0%)	1 (10.0%)	1 (6.3%)
Site of origin				
Enteric flora	2 (28.6%)	4 (25.0%)	10 (100.0%)	16 (100.0%)
Skin flora	4 (57.1%)	5 (31.3%)	0 (0.0%)	0 (0.0%)
*Pseudomonas*	1 (14.3%)	7 (43.8%)	0 (0.0%)	0 (0.0%)
Common pathogen category				
Staphylococcal species	4 (57.1%)	6 (37.5%)	0 (0.0%)	0 (0.0%)
Gram‐negative rod	1 (14.3%)	7 (43.8%)	4 (40.0%)	5 (31.3%)
Opportunistic pathogen	2 (28.6%)	3 (18.8%)	2 (20.0%)	4 (25.0%)
Enterococci	0 (0.0%)	0 (0.0%)	4 (40.0%)	7 (43.8%)

In contrast, MBI‐LCBI infections were consistently dominated by enteric flora, which accounted for 100% of infections in all periods. Most MBI‐LCBI infections from 2010–2016 were Gram‐positive organisms (78.6%), whereas Gram‐negative bacteria predominated in the later period (50.0%). Fungal organisms were identified only in the 2017–2022 MBI‐LCBI group (16.7%). Among pathogen categories, Enterococci were highly represented (50.0% in 2010–2016 and 33.3% in 2017–2022), while Gram‐negative rods increased over time (from 21.4% to 50.0%) (Table 2).

When stratified by prophylaxis status, patients with CLABSI who received FQ prophylaxis had a higher proportion of Gram‐positive infections (71.4%) compared to those without prophylaxis (56.3%). Gram‐negative organisms accounted for 43.8% of CLABSI infections among patients without prophylaxis and 28.6% among those receiving prophylaxis. Notably, *Pseudomonas* was observed in 43.8% of CLABSI cases among patients without FQ prophylaxis but in only 14.3% of those who received prophylaxis. Among MBI‐LCBI cases, Gram‐positive bacteria predominated overall (50.0% in the prophylaxis group and 62.5% without prophylaxis), while fungal infections were infrequent (10.0% and 6.3%, respectively) (Table 3).

Regarding the site of origin, all MBI‐LCBI infections originated from enteric flora (100% across groups), contrasting with CLABSI infections, which were more commonly associated with skin flora (57.1% in the prophylaxis group and 31.3% without prophylaxis). *Pseudomonas* was isolated exclusively among CLABSI cases, predominantly in patients without prophylaxis, reinforcing the observation of fewer Pseudomonal infections in patients receiving FQs. Overall, Staphylococcal species remained the most common CLABSI pathogens, while MBI‐LCBI infections were characterized by Enterococci and Gram‐negative rods (Table 3).

#### 3.2.2 The Role of Prophylaxis in Infections and ICU Admission

In the multivariable logistic regression model evaluating predictors of ICU admission, increasing age was independently associated with higher odds of ICU admission (adjusted odds ratios [aOR] per year 1.03, 95% CI 1.01–1.06). FQ prophylaxis was independently associated with substantially lower odds of ICU admission (aOR 0.22, 95% CI 0.09–0.48). LCBSI, neutropenic fever, C. difficile infection, and TPN use were not independently associated with ICU admission. Model discrimination was acceptable (AUC 0.73, 95% CI 0.65–0.81) (Figure S1 and Table S2).

In the model assessing predictors of LCBSI, increasing age was associated with a modest increase in odds of LCBSI (aOR per year 1.03, 95% CI 1.00–1.05). FQ prophylaxis was associated with numerically lower odds of LCBSI, though the confidence interval crossed unity (aOR 0.54, 95% CI 0.26–1.09). ICU admission, C. difficile infection, and TPN use were not independently associated with LCBSI (AUC 0.63). Similarly, FQ prophylaxis was not independently associated with MBI‐LCBI (aOR 0.68, 95% CI 0.27–1.66) or CLABSI (aOR 0.51, 95% CI 0.19–1.29). No covariates demonstrated independent associations with MBI‐LCBI or CLABSI in adjusted analyses. Discrimination for these models was modest (AUC 0.63 and 0.62, respectively) (Table S2).

## 4 Discussion

In this single‐center retrospective study, we evaluated the real‐world outcomes of FQ prophylaxis among patients undergoing induction chemotherapy for AML. Our results highlight that while FQ prophylaxis did not significantly reduce the incidence of CLABSI or MBI‐LCBI, neutropenic fever, or hospital LOS, it was associated with significantly lower rates of *C. difficile* infection and reduced ICU admissions.

The overall incidence of CLABSI (11.8%) and MBI‐LCBI (13.3%) in our cohort underscores the high burden of infectious complications in patients with AML receiving intensive chemotherapy (Table 1). CLABSIs were increasingly caused by Gram‐positive organisms over time, whereas MBI‐LCBI infections consistently originated from enteric flora, with a temporal shift toward greater Gram‐negative and fungal involvement (Table 2).

When stratified by prophylaxis status, our analysis showed that patients with CLABSI who received FQ prophylaxis were more likely to harbor Gram‐positive organisms (71.4%) compared to those without prophylaxis (56.3%). Gram‐negative bacteria accounted for a greater share of CLABSI infections among patients without prophylaxis. Notably, *Pseudomonas* accounted for approximately one‐third of CLABSI infections overall and was substantially more common among patients who did not receive prophylaxis (43.8% vs. 14.3%), which is consistent with reduced occurrence of this clinically significant pathogen [19, 26] (Table 3).

All MBI‐LCBI infections were derived from enteric flora, consistent with MBI–mediated translocation during profound neutropenia and distinct from catheter‐related bloodstream infections. Historically, many of these infections would have been misattributed to catheter‐related sources under previous CLABSI definitions that did not recognize MBI‐LCBI [12, 13, 15, 16] (Table 3).

Notably, our study contributes new evidence to the field. We found no significant difference in MBI‐LCBI rates between patients who received prophylaxis and those who did not (11.4% vs. 15.0%, *p* = 0.463), suggesting that FQ prophylaxis did not substantially prevent bloodstream infections arising from mucosal translocation during induction chemotherapy in this cohort. This aligns with prior observations indicating that while FQ prophylaxis can reduce infections caused by susceptible organisms, it may have limited effect against translocation events involving endogenous flora [27, 28] (Table 1). This is particularly notable as Gram‐negative and fungal pathogens, which are less reliably prevented by FQs, have become more prominent in MBI‐LCBI over time [29] (Table 3).

Conversely, our observation that ICU admissions were significantly reduced among patients receiving FQ prophylaxis (10.2% vs. 31.8%; OR 0.25, *p* < 0.001) is both novel and clinically relevant. Admission into the ICU for patients with AML is associated with increased morbidity and mortality; therefore the efficacy of prophylactic antibiotics and the relative risk of developing antibiotic resistance is a consideration of utmost importance in the clinical setting [1]. The reduction in ICU utilization suggests that prophylaxis may help prevent infections with highly virulent pathogens such as *Pseudomonas*, leading to fewer instances requiring critical care even in the absence of a significant reduction in total bloodstream infections [19, 30, 31]. Although ICU admissions for sepsis were similar in proportion between patients who received prophylaxis (22.2%) and patients who did not (26.5%), the patients were still too ill for the bone marrow transplant unit. While the mechanisms underlying this finding are not fully clear, it may reflect reduced bacterial load, prevention of early sepsis, or other indirect protective effects of prophylaxis [30] (Table 1).

Patients who received FQ prophylaxis also experienced significantly lower rates of *C. difficile* infection (5.7% vs. 17.8%; OR 0.28, *p* = 0.012). This finding contrasts with earlier concerns that FQ use might increase the risk of *C. difficile* infections [10, 11], but it highlights the utility of this prophylactic practice in the setting of profound neutropenia [21]. Neutropenic fever was frequent in both groups and did not differ significantly (75.0% vs. 84.1%, *p* = 0.113), consistent with previous studies indicating that fever during neutropenia can often arise from non‐infectious causes or infections not preventable by FQ prophylaxis [21, 22]. However, the median duration of time from induction to febrile neutropenia was significantly longer in patients who received FQ prophylaxis (*p *< 0.001), which suggests that FQ prophylaxis may delay the onset of febrile neutropenia, even when it does not fully prevent its occurrence (Table 1). Despite fewer ICU admissions, FQ prophylaxis did not affect ICU or hospital LOS, suggesting its primary benefit may be prevention of early clinical deterioration.

This study has limitations inherent to its retrospective design, including potential selection bias and residual confounding. Our relatively modest sample size may have limited power to detect differences in less common outcomes, and we did not evaluate resistance patterns in detail, which is essential for contextualizing the risks and benefits of FQ use. Nevertheless, our findings contribute valuable real‐world data and help fill gaps in the literature regarding the impact of FQ prophylaxis on MBI‐LCBI and ICU outcomes in AML induction therapy.

In summary, our study demonstrates that while FQ prophylaxis during AML induction chemotherapy does not significantly reduce bloodstream infection rates or hospital LOS, it is associated with lower rates of *C. difficile* infection and significantly fewer ICU admissions. These findings emphasize the need for individualized infection prevention strategies and further research to optimize prophylactic antibiotic use in this vulnerable patient population.

## Author Contributions

C.J.W. was responsible for study conceptualization, methodology, data collection, statistical analysis, interpretation, manuscript drafting, and revisions. S.A., F.B., M.G., C.K., O.I., M.D., A.S., L.D., P.M., and H.L. contributed to data collection. M.M. contributed to statistical analysis and assisted with methodological review. A.C., L.B., J.C., and A.J. provided supervision and critical manuscript review. V.K. contributed to the original research question, supervision, and critical review of the manuscript.

## Funding

The authors have nothing to report.

## Ethics Statement

Ethical review and approval were waived for this study according to federal regulations 45 CFR 46 (DHHS) 2018 Requirements. Decision date: March 29, 2022 with Exemption category # 4c. Secondary research for which consent is not required: secondary research uses of identifiable private information or identifiable biospecimens because the following criteria are met: The research involves only information collection and analysis involving the investigator's use of identifiable health information when that use is regulated under 45 CFR parts 160 and 164, subparts A and E, for the purposes of “health care operations” or “research,” as those terms are defined in 45 CFR 164.501.

## Consent

Due to the retrospective nature of this study, the requirement for informed consent was waived by the Institutional Review Board of Augusta University.

## Conflicts of Interest

J.C. discloses relationships with Novartis, Pfizer, Sun Pharma, Syndax, Nerviano, and Lilly as a consultant. He reports receiving research funding from Novartis, Sun Pharma, Ascentage, and AbbVie. He is also a current holder of stock options and has membership on the Board of Directors at Biopath Holdings. All other authors declare no conflicts of interest.

## Supporting information




**Supporting file 1**: jha270225‐sup‐0001‐SuppMat.docx

## Data Availability

The data that support the findings of this study are available from the corresponding author upon reasonable request.
