# Supplementary material for: Fluoroquinolone Prophylaxis Reduces Intensive Care Unit Admission and Clostridioides difficile Rates in Acute Myeloid Leukemia Induction Chemotherapy
Source: EJHaem. 2026 Feb 15;7(1):e70225. doi: 10.1002/jha2.70225 (PMC12906835; doi:10.1002/jha2.70225)

**Supplementary Table 1: Sample Baseline Characteristics**

| **Characteristic** | **All AML (n=195)** | **Confirmed CLABSI (n=23)** | **Confirmed MBI-LCBI (n=26)** | **p-value** |
| --- | --- | --- | --- | --- |
| Gender (% female) | 85 (43.6%) | 10 (43.5%) | 12 (46.2%) | 0.957¹ |
| Mean Age ± SD (years) | 59.5 ± 13.6 | 60.1 ± 14.3 | 58.9 ± 12.6 | 0.851² |
| Proportion age > 60 years | 106 (54.4%) | 13 (56.5%) | 14 (53.8%) | 0.943¹ |
| Mean Hospital LOS ± SD (days) | 32.8 ± 13.9 | 34.7 ± 15.1 | 33.2 ± 13.2 | 0.676² |
| Received FQ prophylaxis | 88 (45.1%) | 7 (30.4%) | 10 (38.5%) | 0.304¹ |
| CLABSI rate | 23 (11.8%) | 23 (100%) | 0 (0%) | - |
| MBI-LCBI rate | 26 (13.3%) | 0 (0%_ | 26 (100%) | - |
| Febrile neutropenia | 156 (80.0%) | 21 (91.3%) | 22 (84.6%) | 0.275¹ |
| ICU admission | 43 (22.1%) | 5 (21.7%) | 6 (23.1%) | 0.986¹ |
| TPN feeding | 14 (7.8%) | 1 (4.3%) | 3 (11.5%) | 0.612^1^ |
| 60-Day mortality | 8 (4.1%) | 4 (17.4%) | 4 (15.4%) | 0.008^1^ |

¹p-values from chi-square test and ²p-values from ANOVA

**Supplementary Table 2. Multivariable logistic regression analyses of ICU admission and bloodstream infection outcomes during AML induction chemotherapy**

| **Outcome: ICU Admission (events = 43)** |
| --- |
| \| **Predictor** \| **Adjusted OR (95% CI)** \| **Significant** \| \| --- \| --- \| --- \| \| Age (per year) \| 1.03 (1.01–1.06) \| Yes \| \| Fluoroquinolone prophylaxis \| 0.22 (0.09–0.48) \| Yes \| \| LCBSI \| 0.74 (0.31–1.68) \| No \| \| Neutropenic fever \| 0.97 (0.36–2.43) \| No \| \| Clostridioides difficile infection \| 0.85 (0.27–2.34) \| No \| \| Total parenteral nutrition \| 2.57 (0.74–8.60) \| No \| |
| **Outcome: LCBSI (events = 49)** |
| \| **Predictor** \| **Adjusted OR (95% CI)** \| **Significant** \| \| --- \| --- \| --- \| \| ICU admission \| 0.74 (0.31–1.65) \| No \| \| Age (per year) \| 1.03 (1.00–1.05) \| Yes \| \| Fluoroquinolone prophylaxis \| 0.54 (0.26–1.09) \| No \| \| Clostridioides difficile infection \| 0.87 (0.29–2.29) \| No \| \| Total parenteral nutrition \| 1.16 (0.30–3.80) \| No \| |
| **Outcome: MBI-LCBI (events = 26)** |
| \| **Predictor** \| **Adjusted OR (95% CI)** \| **Significant** \| \| --- \| --- \| --- \| \| ICU admission \| 0.79 (0.25–2.15) \| No \| \| Age (per year) \| 1.02 (0.99–1.06) \| No \| \| Fluoroquinolone prophylaxis \| 0.68 (0.27–1.66) \| No \| \| Clostridioides difficile infection \| 1.21 (0.32–3.69) \| No \| \| Total parenteral nutrition \| 1.78 (0.37–6.53) \| No \| |
| **Outcome: CLABSI (events = 23)** |
| \| **Predictor** \| **Adjusted OR (95% CI)** \| **Significant** \| \| --- \| --- \| --- \| \| ICU admission \| 0.76 (0.23–2.18) \| No \| \| Age (per year) \| 1.02 (0.99–1.06) \| No \| \| Fluoroquinolone prophylaxis \| 0.51 (0.19–1.29) \| No \| \| Clostridioides difficile infection \| 0.59 (0.09–2.28) \| No \| \| Total parenteral nutrition \| 0.54 (0.03–3.07) \| No \| |

Adjusted odds ratios (aOR) with 95% confidence intervals are shown. Statistical significance was inferred solely based on whether the confidence interval excluded 1.0.

Supplementary Figure 1:


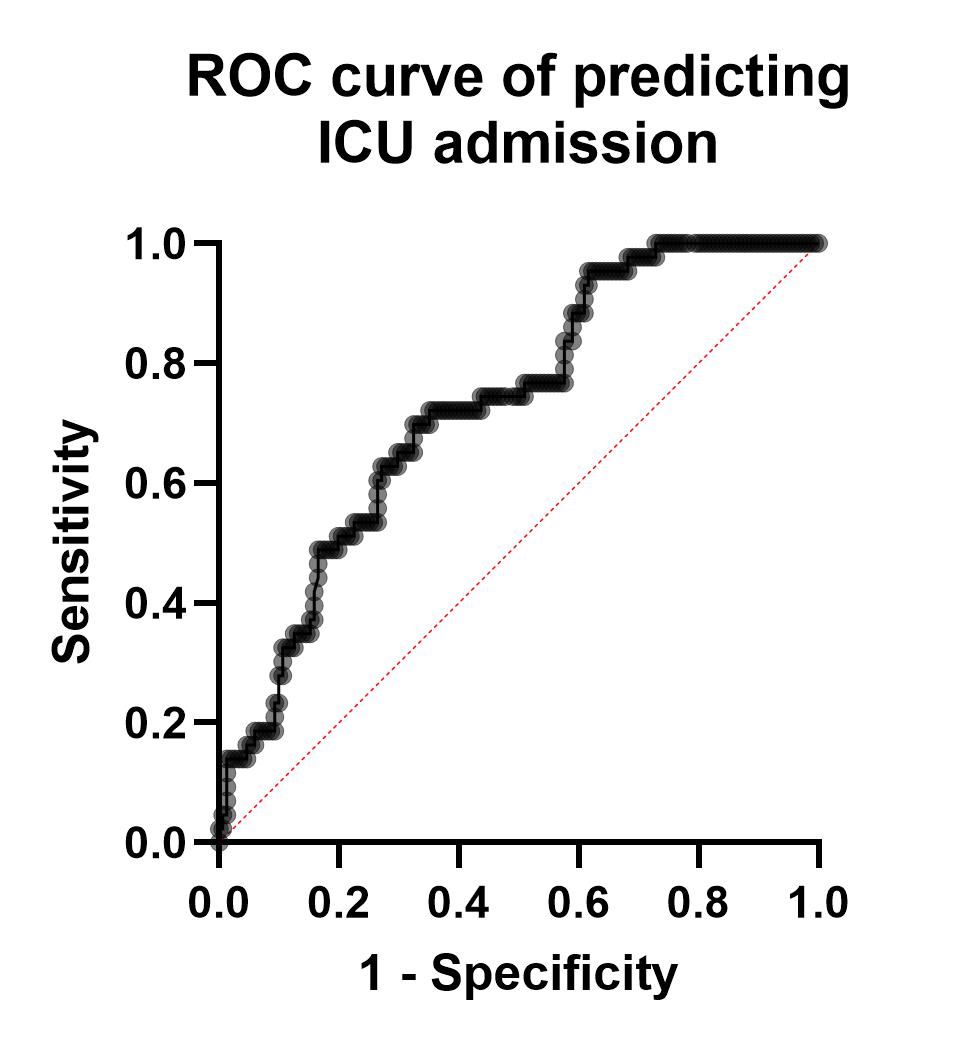

Supplement: Supplementary file 1 — Supporting file 1: jha270225‐sup‐0001‐SuppMat.docx [file JHA2-7-e70225-s001.docx]
